# Nox‐4 deletion reduces oxidative stress and injury by PKC‐*α*‐associated mechanisms in diabetic nephropathy

**DOI:** 10.14814/phy2.12192

**Published:** 2014-11-04

**Authors:** Vicki Thallas‐Bonke, Jay C. Jha, Stephen P. Gray, David Barit, Hermann Haller, Harald H.H.W. Schmidt, Melinda T. Coughlan, Mark E. Cooper, Josephine M. Forbes, Karin A.M. Jandeleit‐Dahm

**Affiliations:** 1 Diabetes Complications Division, Baker IDI Heart & Diabetes Institute, JDRF Danielle Alberti Memorial Centre for Diabetic Complications, Melbourne, Victoria, Australia; 2 Department of Medicine, Austin and Northern Clinical Schools, University of Melbourne, Melbourne, Victoria, Australia; 3 Department of Medicine, Central Clinical School, Monash University, AMREP Precinct, Melbourne, Victoria, Australia; 4 Department of Nephrology and Hypertension, Hannover Medical School, Hannover, Germany; 5 Pharmacology, Faculty of Health, Medicine & Life Sciences, Cardiovascular Research Institute Maastricht (CARIM), Maastricht University, Maastricht, The Netherlands; 6 Department of Epidemiology & Preventive Medicine, Monash University, AMREP Precinct, Melbourne, Victoria, Australia; 7 Mater Medical Research Institute, School of Medicine, University of Queensland, South Brisbane, Queensland, Australia

**Keywords:** Diabetic nephropathy, NADPH oxidase, protein kinase C, reactive oxygen species

## Abstract

Current treatments for diabetic nephropathy (DN) only result in slowing its progression, thus highlighting a need to identify novel targets. Increased production of reactive oxygen species (ROS) is considered a key downstream pathway of end‐organ injury with increasing data implicating both mitochondrial and cytosolic sources of ROS. The enzyme, NADPH oxidase, generates ROS in the kidney and has been implicated in the activation of protein kinase C (PKC), in the pathogenesis of DN, but the link between PKC and Nox‐derived ROS has not been evaluated in detail in vivo. In this study, global deletion of a NADPH‐oxidase isoform, Nox4, was examined in mice with streptozotocin‐induced diabetes (C57Bl6/J) in order to evaluate the effects of Nox4 deletion, not only on renal structure and function but also on the PKC pathway and downstream events. Nox4 deletion attenuated diabetes‐associated increases in albuminuria, glomerulosclerosis, and extracellular matrix accumulation. Lack of Nox4 resulted in a decrease in diabetes‐induced renal cortical ROS derived from the mitochondria and the cytosol, urinary isoprostanes, and PKC activity. Immunostaining of renal cortex revealed that major isoforms of PKC, PKC‐*α* and PKC‐*β*1, were increased with diabetes and normalized by Nox4 deletion. Downregulation of the PKC pathway was observed in tandem with reduced expression of vascular endothelial growth factor (VEGF), transforming growth factor (TGF)‐*β*1 and restoration of the podocyte slit pore protein nephrin. This study suggests that deletion of Nox4 may alleviate renal injury via PKC‐dependent mechanisms, further strengthening the view that Nox4 is a suitable target for renoprotection in diabetes.

## Abstract (precis)

Diabetic nephropathy is an increasing problem with current treatments unable to stop the disease. Nox‐4 is implicated as a major source of reactive oxygen species in the kidney, both cytosolic and mitochondrial. Global deletion of Nox‐4 in a streptozotocin model of diabetes was able to attenuate a number of important renal functional and structural parameters including albuminuria and extracellular matrix accumulation. The importance of these findings is the link between Nox‐4 and PKC‐*α* activation leading to diabetic nephropathy.

## Introduction

The prevalence of DN rises each year (Shaw et al. 2010), resulting in an increased number of individuals with end‐stage renal disease worldwide. Hyperglycemia drives the progression of diabetic complications including DN. A number of mechanisms have been implicated in the tissue‐damaging effects of hyperglycemia, such as the polyol pathway (Yabe‐Nishimura 1998), increased production of advanced glycation end‐products (Brownlee et al. 1988), activation of PKC isoforms (Lee et al. 1989), and oxidative stress (Baynes and Thorpe 1999). A unifying hypothesis has been postulated which connects these diverse mechanisms (Brownlee 2005) suggesting that excess generation of mitochondrial superoxide by hyperglycemia is the primary initiating factor linking these pathways to tissue damage.

However, the key source of ROS within the cells of the kidney in the diabetic milieu is not fully established. Mitochondria are thought to be a principal generator of ROS derived from the respiratory chain in diabetes (Nishikawa et al. 2000; Coughlan et al. 2009). More recent studies have postulated that NADPH oxidases are the key source of ROS production in diabetes (Sedeek et al. 2010; Gorin and Block 2013). NADPH oxidase is composed of two membrane‐associated components, p22phox and gp91phox, and four major cytosolic components, p47phox, p40phox, p67phox, and rac‐1/2. The gp91phox component has a number of other homologues, now referred to as the Nox family of NADPH oxidases, (Nox1‐5 and Duox 1‐2) which are able to reduce superoxide into oxygen by transporting electrons across membranes (Bedard and Krause 2007). This Nox family of enzymes has also been implicated in other cell functions including cell proliferation, host defense, differentiation, apoptosis, senescence, gene expression, oxygen sensing, angiogenesis, and cellular signaling (Bedard and Krause 2007).

Nox1, Nox2, and Nox4 (Gill and Wilcox 2006) and more recently Nox5 (Holterman et al. 2013) have been implicated in DN. Nox4 has been shown to be a major source of ROS in renal cells including vascular smooth muscle cells, endothelial cells, podocytes, and epithelial cells of various tubules of the kidney in diabetic animals (Zou et al. 2001; Asaba et al. 2005; Wendt et al. 2005; Xia et al. 2006). A study by Block et al. (2009) found that Nox4 localizes to membranes and also to the mitochondria in the renal cortex in diabetes.

Thus, the aim of this study was to examine the renoprotective role of deletion of Nox4 and in particular to examine potential downstream mediators of renal injury that are attenuated upon loss of Nox4 signaling, in particular, with a focus on cytosolic and mitochondrial ROS generation as well as the evaluation of PKC‐*α* and PKC‐*β*, which mediate various functional and structural features of DN.

Thus, the aim of this study was to examine the renoprotective role of deletion of Nox4 and in particular to examine potential downstream mediators of renal injury that are attenuated upon loss of Nox4 signaling. In particular, novelty was evaluation of PKC‐*α* and PKC‐*β* and the link to Nox4 activation in an in vivo model of DN.

## Materials and Methods

### Experimental animal model

Male wild‐type C57BL6/J mice and mice with a genetic deletion of Nox4 on a C57BL6/J background (Nox4‐KO) were obtained from Prof Harald Schmidt (Kleinschnitz et al. 2010). Experimental diabetes was induced in male WT (D), and Nox‐4 (KO‐D) (*n *=**10/group) mice by intraperitoneal injection of streptozotocin (STZ) at a dosage of 55 mg/kg daily for five consecutive days as described previously (mice with blood glucose >15 mmol/L were included as diabetic) (Forbes et al. 2004). Control (WT‐C) and Nox4 control (KO‐C) (*n *=**10/group) mice were injected with vehicle (sodium citrate buffer, pH 4.5). Water and food were provided ad libitum. At 20 weeks of the study, a blood sample was collected prior to the mice being housed in metabolic cages, for a 24‐h urine collection. Glycated hemoglobin (GHb) was determined by HPLC (CLC330 GHb Analyzer; Primus, Kansas City, MO) (Cefalu et al. 1994) and urinary albumin by enzyme‐linked immunosorbent assay (ELISA) (Bethyl Laboratories, Montgomery, TX) (Coughlan et al. 2007b). Mean systolic blood pressure was estimated by a computerized, noninvasive tail cuff plethysmography in conscious mice at week 20 (Krege et al. 1995).

All groups of mice were followed up for 20 weeks. Animal procedures were in accordance with guidelines provided by the AMREP (Alfred Medical Research and Education Precinct, Ethics number E/0977/2010/B), Animal Ethics Committee and the National Health and Medical Research Council of Australia.

### qRT‐PCR

Total RNA was extracted after homogenizing renal cortex (Polytron PT‐MR2100; Kinematicca, Littau/Lucerne, Switzerland) in TRIzol reagent (Invitrogen Australia, Mt Waverly, Vic, Australia) as described previously (Jha et al. 2014). Gene expression using a probe for the Nox4 primers sequence was as follows: forward primer (5′‐GGCTGGAGGCATTGGAGTAA‐3′) and reverse primer (5′‐ CCAGTCATCCAACAGGGTGTT‐3′). Data were analyzed quantitatively and relative to the expression of the housekeeping gene 18S (18S ribosomal RNA Taqman Control Reagent kit) using Taqman© system (ABI Prism 7500; Perkin‐Elmer, Poster City, CA) (Jha et al. 2014). Results are expressed relative to nondiabetic Nox4 mice, which were arbitrarily assigned a value of 1.

### Genotyping

Genomic DNA was isolated from tail biopsies of mice using Maxwell^®^ 16 DNA Purification Kits (Promega Corporation, Madison, WI) and used subsequently for routine genotype analysis using a PCR method of GoTaq^®^ DNA polymerase and gel electrophoresis from Promega. The following primer sequences were used for genotyping: WT‐Nox4 forward 5′GTG GAT CAA GAA ACA TGC TGA C 3′and WT‐Nox4 reverse 5′AGA CAT CCA ATC ATT CCA GTG G 3′ The band sizes of the PCR products of the Nox4 WT mice obtained were Nox4WT (435 bp).

### Urinary creatinine

Urinary creatinine was determined by a commercially available creatinine assay kit according to the manufacturer's instructions (Abcam, Cambridge, UK). Fluorescence was determined by a FLUOstar Omega plate reader (BMG LABTECH, Ortenberg, Germany) with Ex/Em = 538/587 nm. Data were normalized to 24‐h urine volume.

### Cystatin C

Plasma cystatin C was determined according to the manufactures instructions using a mouse Cystatin C ELISA kit (BioVendor, Brno, Czech Republic). The absorbance was measured at 450 nm by a Victor 3V multilabel plate reader.

### Histological assessment of kidney injury

Kidney sections were stained with periodic acid Schiff's stain for quantitation of the glomerulosclerotic index (GSI). The degree of glomerulosclerosis (20 glomeruli/kidney), which was defined as thickening of the basement membrane and mesangial expansion, was evaluated by a semiquantitative method as described previously (Giorgi et al. 2010).

### Renal cortical fractionation

Kidney cortex was homogenized in extraction buffer (20 mmol/L HEPES, 1 mmol/L EDTA, 10 mmol/L EGTA, 210 mmol/L sucrose, pH 7.2) containing complete protease (Roche Diagnostics, Basel, Switzerland) and phosphatase inhibitors (Sigma, St Louis, MO). The homogenates were centrifuged at 1000 *g* for 10 min liberating the nuclear fraction in the pellet. The resulting supernatants were centrifuged at 10,000 g at 4°C for 20 min with the resulting pellets containing the mitochondrial. The supernatants were then ultracentrifuged at 100,000 g for 1 h at 4°C. This supernatant was retained as the cytosolic fraction and the pellet was resuspended in extraction buffer (with 1%Triton X‐100) and ultracentrifuged at 100,000 g for 1 h at 4°C. This supernatant was retained as the membranous fraction (Thallas‐Bonke et al. 2013).

### Superoxide production

Tissue superoxide production was assessed in renal cortex as follows. Kidneys were rapidly excised, placed in oxygen‐saturated Krebs buffer, and cut into ~1 mm^3^ segments (Coughlan et al. 2007a). The rate of superoxide anion formation in the kidney was determined by chemiluminescence of lucigenin (bis‐*N*‐methylacridinium nitrate; Sigma Chemical Company) using a luminometer (Microlumat Plus, Berthold Technologies, Wildbad, Germany) as described previously. Results expressed as relative light units were normalized to 10‐mg dry tissue weight as described previously (Coughlan et al. 2009).

### 8‐Iso‐PGF 2a ELISA

The Urinary Isoprostanes EIA kit (Oxford Biomedical Research, Oxford, MI) was used to measure 15‐isoprostanes F_2t_ in urine as described by the manufacturer. Urinary 15‐isoprostanes F_2t_ concentrations were standardized to 24‐h urine volume to obtain excretion over 24 h.

### VEGF ELISA

The Quantikine Mouse ELISA kit (R&D Systems, Inc., Minneapolis, MN) was used to measure VEGF in the urine as per the kit instructions and expressed as pg per 24 h (Thallas‐Bonke et al. 2010).

### TGF‐β1 ELISA

Transforming growth factor‐*β* protein was measured in urine and renal cortical membranous fraction. Biologically active TGF‐*β*1 was measured by the TGF‐*β*1 E_max_® ImmunoAssay System (Promega). Prior to the addition of samples to the ELISA plate, the active form of TGF‐*β*1 was liberated by acid treatment of samples with 1N HCl followed by the neutralization with 1N NaOH as per the manufacturer's instructions. Values were expressed as pg/24 h (urine) or pg/mg protein (plasma membrane) as determined by the BCA protein assay (Perbio Science UK, Cheshire, UK) (Thallas‐Bonke et al. 2010).

### Immunohistochemistry

Immunohistochemistry was performed as described previously (Thallas‐Bonke et al. 2008). In brief, slides for collagen IV and fibronectin required digested with 0.4% pepsin at 37°C. Kidney sections for PKC‐*α*, PKC‐*β*1, and nitrotyrosine did not require antigen retrieval and sections for nephrin required antigen retrieval. The primary antibodies were goat anti‐human collagen IV (1:500; Southern Biotech, Birmingham, AL), rabbit anti‐fibronectin (1:800, Dako Cytomation, Glostrup, Denmark), rabbit anti‐human PKC‐*α*, rabbit anti‐human PKC‐*β*1 and goat anti‐human nephrin (1:100, 1:100, 1:500, Santa Cruz Biotechnology, Cambridge, UK), and rabbit anti‐nitrotyrosine (1:100, Millipore, Billercia, MA). Quantitation of renal cortical immunohistochemistry was performed by computer‐aided densitometry (Optimus 6.5; Media Cybernetics, Silver Springs, MD), as described previously (Thallas‐Bonke et al. 2008).

### PKC activity

Membrane, mitochondrial, nuclear, and cytosolic fractions were assayed for PKC activity using the StressXpress PKC Kinase ActivityAssay Kit (EKS‐420A; Stressgen Bioreagents, Victoria, BC, Canada) as described previously (Coughlan et al. 2007b). Data were normalized to total protein content, as measured by the BCA assay.

### PKC‐α ELISA

Protein kinase C‐*α* protein was determined in the membranous and mitochondrial renal cortical fractions using a commercial competitive ELISA kit (MyBioSource, San Diego, CA) as per the manufacturer's instructions. PKC‐*α* concentration was interpolated from a standard curve and expressed as ng/mg protein.

### Statistical analysis

All statistical computations were performed using GraphPad Prism version 4.0a for Mac OS X (GraphPad Software, San Diego, CA). Results are expressed as mean ± standard deviation unless otherwise specified. Analyses were performed by ANOVA followed by the post‐hoc analysis using Tukey's test. Data for ACR were not normally distributed and log transformed prior to analysis. A probability of *P *<**0.05 was considered statistically significant.

## Results

### Nox4 deletion

**Figure 1.**
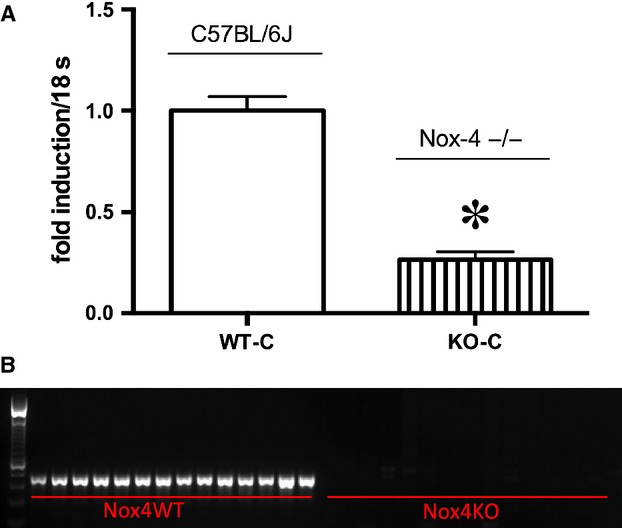
(A) Analysis of Nox4 mRNA levels in the renal cortex of control wild‐type and knockout mice. B) Genotyping by PCR of genomic DNA extracted from mouse tails of wild‐type and Nox4 knockout mice.

Deletion of Nox4 was confirmed by the analysis of Nox4 mRNA levels in the renal cortex of kidneys and by genotyping by PCR of genomic DNA extracted from mouse tails (Fig. 1).

### Physiological and functional parameters

Induction of diabetes was associated with decreased body weight and increased kidney mass to body surface area ratio in both groups (Table 1). Mean systolic blood pressure was unaffected by induction of diabetes or deletion of Nox4 (Table 1). Glycated hemoglobin and plasma glucose were significantly increased by diabetes, independent of deletion of Nox4 (Table 1).

**Table 1.** Physiological and structural parameters of mice at week 20 of the study. Data are shown as Mean ± SD, except for AER shown as Mean ± SEM

	Control		Diabetic	
	WT (20 weeks)	Nox‐4 KO (20 weeks)	WT (20 weeks)	Nox‐4 KO (20 weeks)
Body weight (g)	37.2 ± 2.9	37.1 ± 2.1	24.6 ± 2.8*	26.1 ± 3.7†
Kidney mass/BSA (g/m^2^)	35.1 ± 3.8	33.4 ± 3.4	52.6 ± 6.0*	51.5 ± 4.5†
Systolic BP (mmHg)	109 ± 8	97 ± 9	100 ± 14	98 ± 13
Glycated hemoglobin (%)	3.9 ± 0.4	3.9 ± 0.9	14.1 ± 2.2*	12.0 ± 5.4†
Plasma glucose (mmol/L)	11 ± 2	10 ± 2	28 ± 6*	26 ± 6†

BSA, body surface area.

* *P *<**0.001 versus WT‐C.

† *P *<**0.001 versus KO‐C; *n *=**15/group.

**Figure 2.**
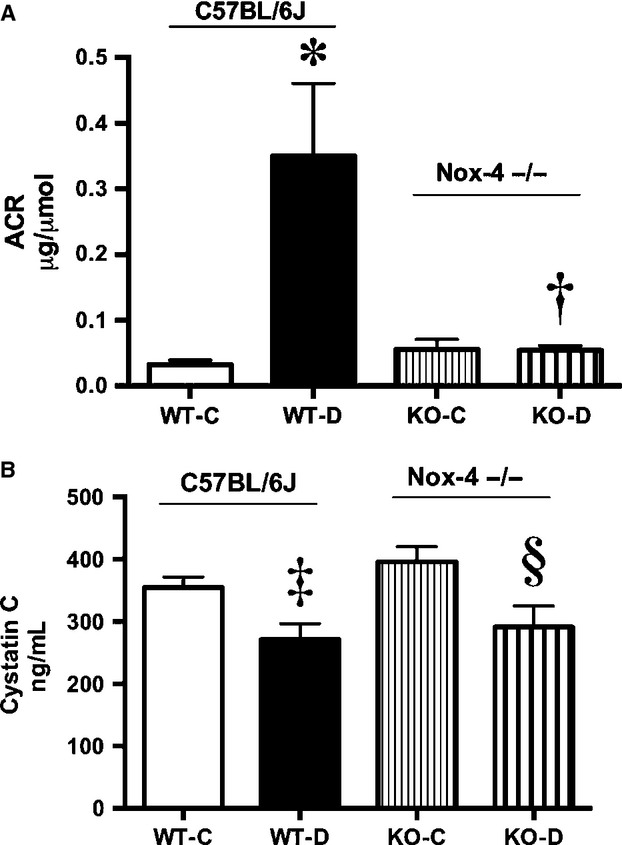
Renal functional data at week 20. A) Urinary albumin to creatinine ratio (ACR), B) Plasma cystatin C, *P <**0.01 versus WT‐C; ^†^*P *<**0.05 versus WT‐D; ^‡^*P *<**0.05 versus WT‐C; ^§^*P *<**0.05 versus KO‐C group, *n *=**8–10/group.

**Figure 3.**
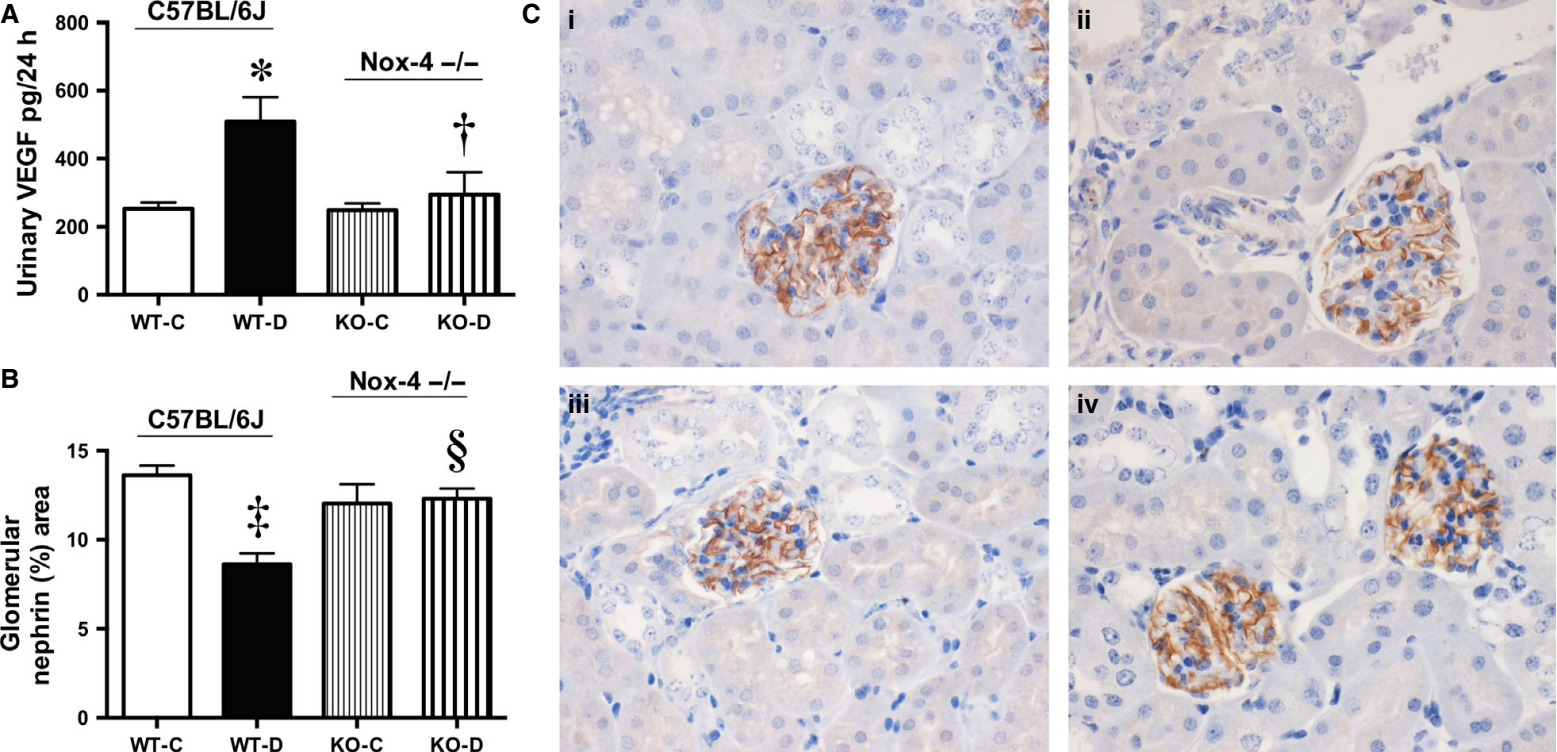
Renal parameters at 20 weeks. A) Urinary VEGF excretion. B) Computer‐aided analysis of immunohistochemical staining of glomerular nephrin expression, C) Representative photomicrographs of nephrin immunohistochemical staining of renal cortex, (i) WT‐C; (ii) WT‐D, (iii) Nox4‐KO‐C; (iv) Nox4‐KO‐D, *P <**0.01 versus WT‐C; ^†^*P *<**0.05 versus WT‐D; ^‡^*P *<**0.001 versus WT‐C; ^§^*P *<**0.01 versus WT‐D, *n *=**8–10/group; all scale bars 50 *μ*m.

The diabetes‐induced increase in albuminuria in wild‐type diabetic (WT‐D) mice was ameliorated in the Nox4−/− diabetic mice (KO‐D) (Fig. 2A). Plasma cystatin C, regarded as a more sensitive marker of glomerular filtration rate than serum creatinine (Dharnidharka et al. 2002), was decreased in both WT and Nox4−/− diabetic mice (Fig. 2B) indicating that hyperfiltration was not normalized by Nox4 deletion. Albuminuria has been previously linked to VEGF expression including diabetes (Sung et al. 2006; Ziyadeh and Wolf 2008). Indeed, there was an increase in urinary VEGF in WT diabetic mice (Fig. 3A), which was normalized in Nox4−/− mice. There was a concomitant decrease in the expression of glomerular nephrin in the diabetic mice, which was also abrogated in the Nox4 KO‐D mice (Fig. 3B and C).

### Structural parameters – extracellular matrix proteins

**Figure 4.**
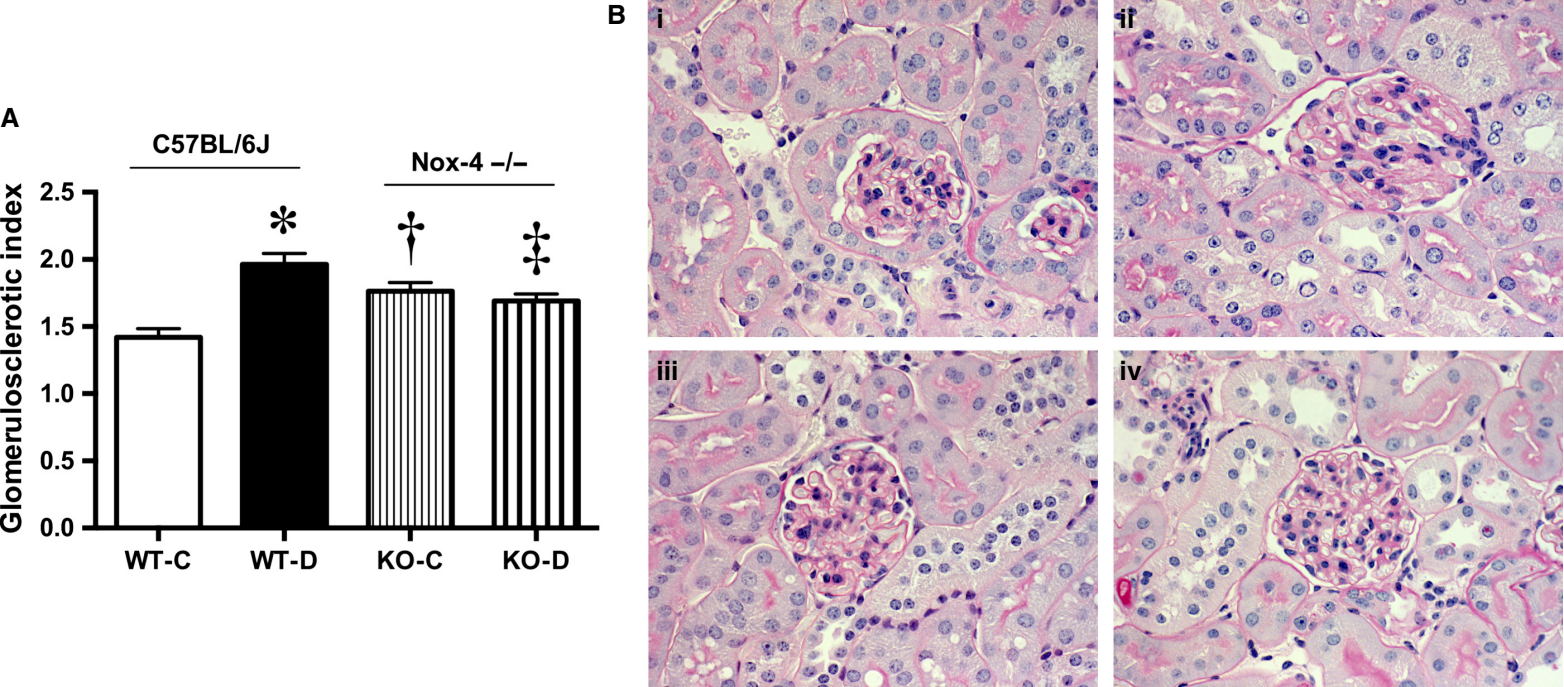
Renal structural data at week 20. A) Glomerulosclerotic index (GSI), B) Representative photomicrographs of PAS stained renal cortex of kidney (×400 magnification), (i) WT‐C; (ii) WT‐D, (iii) Nox4‐KO‐C; (iv) Nox4‐KO‐D, *P <**0.001 versus WT‐C; ^†^*P *<**0.05 versus WT‐C and ^‡^*P *<**0.05 versus WT‐D, *n *=**8–10/group.

**Figure 5.**
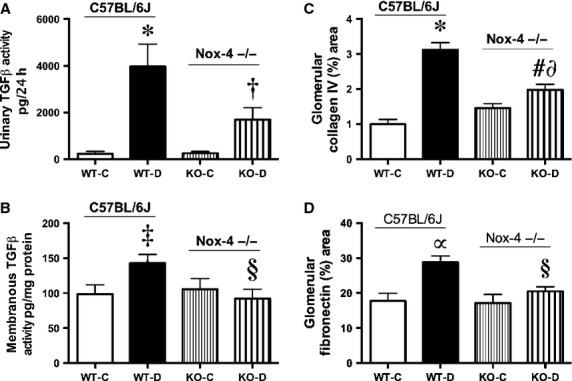
Various renal parameters at week 20, A) Biologically active TGF‐*β*1 excretion, B) Biologically active membranous TGF‐*β*1 expression, C) Computer‐aided image analysis of the extracellular matrix protein collagen IV by immunohistochemistry, expressed as percent area in glomeruli, D) Computer‐aided image analysis of the extracellular matrix protein fibronectin by immunohistochemistry, expressed as percent area in glomeruli, *P <**0.001 versus WT‐C; ^†^*P *<**0.01 versus WT‐D; ^‡^*P *<**0.05 versus WT‐C; ^§^*P *<**0.05 versus WT‐D; ^#^*P *<**0.001 versus WT‐D; ^∂^*P *<**0.05 versus KO‐C; *^α^P* < 0.01 versus WT‐C, *n *=**8–10/group.

The GSI, a measure of glomerular damage, was increased with diabetes in the Nox4 WT‐D mice. Nox4 KO‐D mice showed improved GSI compared to WT‐D mice (Fig. 4A and B). Induction of diabetes was associated with an increase in biologically active TGF‐*β*1 in urine, which was partly, but not fully, reduced in the Nox4 KO‐D mice (Fig. 5A). Further analysis of renal cortical plasma membrane fractions of the diabetic Nox4 deleted mice revealed that the expression of TGF‐*β*1 activity was normalized compared to the WT‐D mice (Fig. 5B). Consistent with the levels of the prosclerotic cytokine TGF‐*β*, the protein expression of renal glomerular collagen IV was increased in diabetic WT mice. Furthermore, the Nox4 KO‐D mice had decreased protein expression compared to their WT‐D counterparts. However, Nox4 deleted control mice had significantly lower collagen IV expression when compared to the respective KO‐D mice (Fig. 5C). The extracellular matrix protein fibronectin was also increased in the WT‐D mice and significantly ameliorated with Nox4 deletion (Fig. 5D).

### Oxidative stress

**Figure 6.**
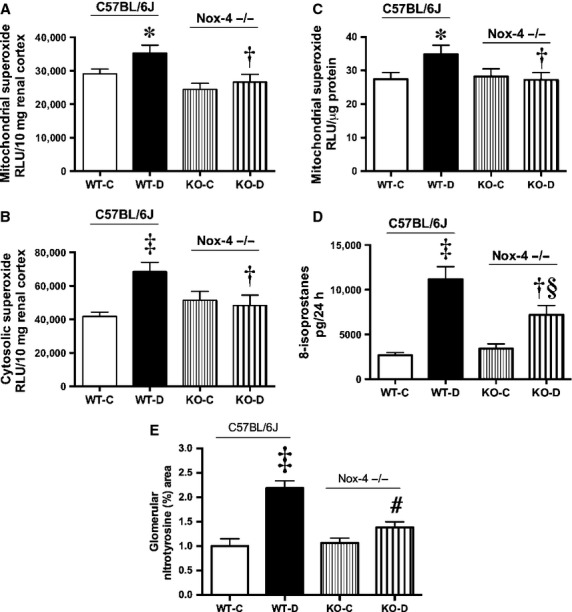
Oxidative stress markers at 20 weeks from renal cortex, A) Ex vivo NADH‐dependent mitochondrial tissue superoxide production, B) Ex vivo NADPH‐dependent cytosolic tissue superoxide production, C) Superoxide production from isolated mitochondria, D) Urinary 8‐isoprostane excretion, E) Computer‐aided analysis of immunohistochemical staining of renal cortical nitrotyrosine expressed as percent area, *P <**0.05 versus WT‐C; ^†^*P *<**0.05 versus WT‐D; ^‡^*P *<**0.001 versus WT‐C; ^§^*P *<**0.05 versus KO‐C, ^#^*P *<**0.01 versus WT‐D, *n *=**8–10/group.

It has been suggested that mitochondria are a major source of ROS, which may play a role in the pathogenesis of DN (Forbes et al. 2008). Furthermore, Nox4 has recently been reported to localize within mitochondria (Block et al. 2009) but if Nox4 is a major source of mitochondrial ROS generation in diabetes remains unknown. Therefore, we measured superoxide production in live renal cortical tissue slices and in isolated mitochondria in Nox4 replete and KO mice in the presence and absence of diabetes. There was excess of superoxide production from both renal cortical mitochondrial (NADH‐driven) and cytosolic (NADPH‐driven) sources in diabetes (Fig. 6A and B), with more superoxide derived from the cytosolic compartment. Deletion in Nox4 resulted in normalization of superoxide production, suggesting that NADPH oxidase directly impact on mitochondrial superoxide production within the renal cortex. This was verified in isolated mitochondria (Fig. 6C). However, assessment of “whole body” superoxide production, by measurement of a stable marker of lipid peroxidation, 15‐isoprostane F_2t_ showed residual oxidative stress status subsequent to Nox4 deletion (Fig. 6D), suggesting that there remain sources of oxidative stress that are independent of NADPH oxidase. Immunohistochemical evaluation of another oxidative stress marker, nitrotyrosine in glomeruli demonstrated a diabetes‐induced increase in the wild‐type mice, which was reduced in the diabetic KO mice (Fig. 6E).

### PKC

**Figure 7.**
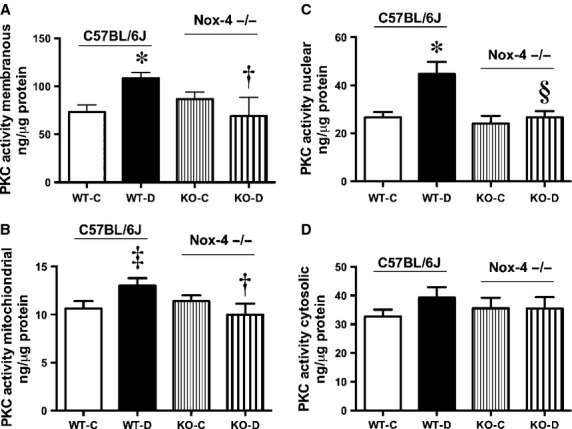
PKC activity of subcellular renal cortical compartments from 20‐week‐old mice, A) Membranous, B) Mitochondrial, C) Nuclear, D) Cytosolic, *P <**0.01 versus WT‐C; ^†^*P *<**0.05 versus WT‐D; ^‡^*P *<**0.05 versus WT‐C; ^§^*P *<**0.01 versus WT‐D; *n *=**8–10/group.

Activation of PKC has been implicated in the pathogenesis of DN and the induction of mitochondrial ROS in response to glucose has been confirmed in mesangial cells (Kiritoshi et al. 2003); however, the link to subsequent PKC activation has been less well characterized. Thus, we measured PKC activity in specific cellular compartments of the renal cortex from mice with and without expression of Nox4. Diabetes induced an increase in PKC activity in the plasma membrane (Fig. 7A), mitochondria (Fig. 7B), and nucleus (Fig. 7C). This activity was normalized by Nox4 deletion. The PKC activity in the cytosolic fraction was unaffected (Fig. 7D).

**Figure 8.**
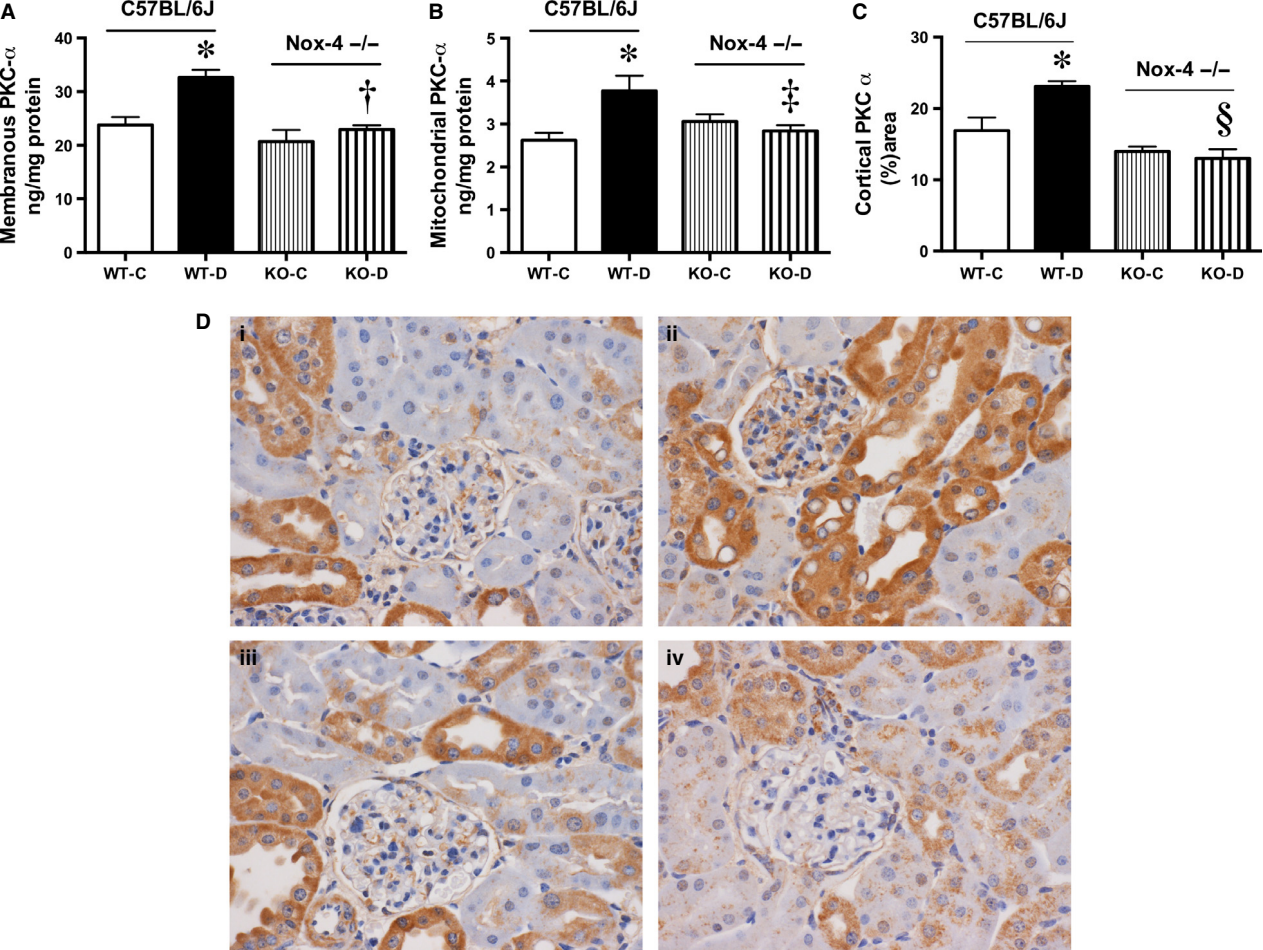
PKC‐*α* expression at 20 weeks A) Membranous fraction and B) Mitochondrial fraction of renal cortex by ELISA. C) Computer‐aided analysis of immunohistochemical staining of renal cortical PKC‐*α* expressed as percent area and D) Representative photomicrographs of PKC‐*α* immunostaining in renal cortex of kidney (×400 magnification), (i) WT‐C; (ii) WT‐D, (iii) Nox4‐KO‐C; (iv) Nox4‐KO‐D. *P <**0.01 versus WT‐C; ^†^*P *<**0.01 versus WT‐D; ^‡^*P *<**0.05 versus WT‐D, ^§^*P *<**0.001 versus WT‐D, *n *=**8–10/group.

**Figure 9.**
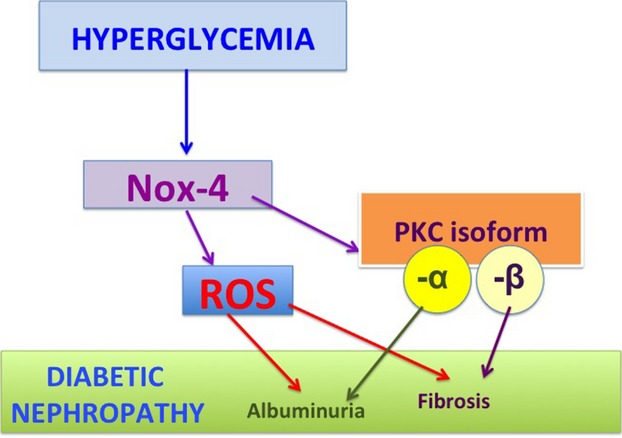
PKC‐*β*1 expression at 20 weeks, A) Computer‐aided analysis of immunohistochemical staining of renal cortical PKC‐*β*1 expressed as percent area (*n *=**8–10/group), B) Representative photomicrographs of PKC‐*β*1 immunostaining in renal cortex of kidney (×400 magnification), (i) WT‐C; (ii) WT‐D, (iii) Nox4‐KO‐C; (iv) Nox4‐KO‐D. *P <**0.001 versus WT‐C; ^†^*P *<**0.001 versus WT‐D.

Next, the key canonical isoforms of PKC were determined. PKC‐*α* content of the plasma membrane (Fig. 8A) and the mitochondria (Fig. 8B) was increased in diabetic mice, and this increase was attenuated by Nox4 deletion. An overall assessment of PKC‐*α* in the renal cortex by immunohistochemical staining demonstrated an increase in the WT‐D mice compared to the WT‐C with a significant decrease in the Nox4 KO‐D animals (Fig. 8C and D). Similar results were observed with PKC‐*β*1, in which an increase in protein expression was found in the WT‐D group with a significant reduction in the KO‐D animals (Fig. 9A and B).

## Discussion

**Figure 10.**
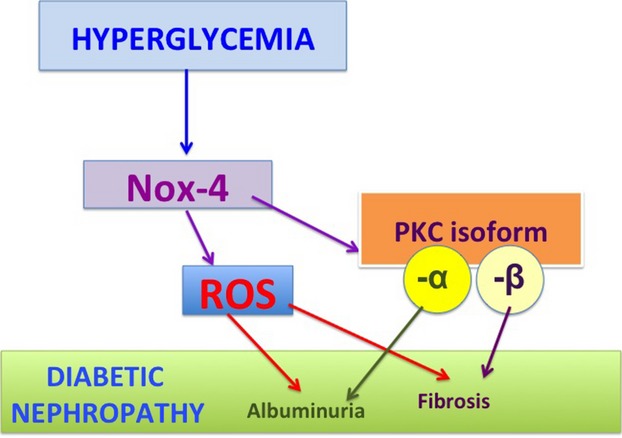
Speculative schema for the involvement of Nox4 and PKC in diabetic nephropathy.

These studies have focused on the role of deletion of Nox4 on the regulation of the PKC pathway in diabetic kidney disease (Fig. 10). We have shown for the first time the renoprotective effects of global deletion of Nox4 in a model of streptozotocin‐induced diabetes. This resulted in improved renal injury with significant reduction in albuminuria, GSI, mitochondrial and cytosolic sources of superoxide, a decrease in urinary isoprostanes, and expression of glomerular collagen IV and fibronectin in association with reduced expression of the PKC‐*α* and ‐*β* isoforms. Previous studies in Nox4 knockout mice have produced conflicting data (Babelova et al. 2012), with our recent study showing a renoprotective effect of Nox4 deletion in a different model (Jha et al. 2014). However, the mechanisms whereby Nox4 appears to afford renoprotection and in particular the link to PKC remain inadequately examined and need further evaluation.

It is widely appreciated that ROS are critical for cell signaling as well as driving immune‐mediated processes via the oxidative burst, which is primarily mediated by NADPH oxidase (Droge 2002). However, excess production or reduced clearance of ROS resulting in persistent oxidative stress can often be deleterious and is implicated in the pathogenesis of chronic diseases such as diabetes and its complications including nephropathy (Duchen 2004; Brownlee 2005; Forbes et al. 2008). The importance of Nox4 is supported by previous studies, which demonstrated that administration of an antisense Nox4 cDNA to rats can prevent the development of DN (Gorin et al. 2005). Recent studies by our group using the ApoE/Nox4 double KO mouse, a model with the superimposition of excess lipid, demonstrated reduced albuminuria in association with decreased renal morphological injury in these double KO diabetic mice (Jha et al. 2014). Indeed, Nox4 deletion led to reduced renal injury, as assessed at both a functional and structural level. This attenuation in renal injury was associated with reduced intrarenal ROS. Furthermore, this link between Nox4 deletion and renal oxidative stress appears to be functionally relevant, since the diabetic Nox4 KO mice have reduced albuminuria.

The present findings contrast with another study (Babelova et al. 2012) that only examined mice for a relatively short duration of 8 weeks, those animals having more modest injury than observed in this study where duration of diabetes was 20 weeks. Other groups have also reported studies consistent with Nox4‐promoting renal injury by different approaches rather than gene deletion to decrease Nox4 activity. These include an antisense approach (Gorin et al. 2005) and the use of pharmacological inhibitors (Sedeek et al. 2010), both studies indicating that decreased Nox4 activation confers renal benefits in the setting of experimental diabetes.

Previous studies by our group have demonstrated that excess mitochondrial superoxide generation observed in the diabetic kidney is not affected by current clinically used therapies such as ACE inhibitors (Coughlan et al. 2007b). In contrast, other interventions including an inhibitor of AGE accumulation, deletion of the AGE receptor RAGE, and a nonspecific antioxidant apocynin were all shown to inhibit ROS, of both mitochondrial and cytosolic origin (Thallas‐Bonke et al. 2008, 2013; Tan et al. 2009) in association with improved renal injury. The disparate effects of antioxidants such as apocynin and ACE inhibitors on mitochondrial ROS appear to be therapeutically relevant since a combination of apocynin and ramipril was more effective than either monotherapy (Thallas‐Bonke et al. 2010).

We have also previously shown that an interaction between cytosolic and mitochondrial sources of ROS amplifies kidney disease in diabetes (Coughlan et al. 2009). This link is further supported by a recent report demonstrating that Nox4 is localized to mitochondria (Block et al. 2009). Indeed, in this study, we show that the increase in mitochondrial ROS in the diabetic kidney is not observed in the absence of Nox4, a finding consistent with recent reports of the presence of Nox4 in the mitochondria. It remains to be determined if Nox4 directly modulates mitochondrial ROS from within the mitochondrial membranes, for example, at the level of the respiratory chain, or by influencing other ROS‐producing enzymes. Another possibility is that Nox4 regulates cytosolic ROS leading to increased mitochondrial ROS production via opening of the mitochondrial permeability transition pore, as reported by our group previously (Coughlan et al. 2009). In this study, an increase in urinary 15‐isoprostane F_2t_ and renal nitrotyrosine expression as seen in diabetic mice is consistent with an overall increase in renal oxidative stress. However, there was a persistent increase in 15‐isoprostane F_2t_ expression consistent with residual oxidative stress status subsequent to Nox4 deletion, thereby suggesting that there remain other sources of oxidative stress in the diabetic setting that are independent of NADPH oxidase.

Seminal studies from Brownlee's group demonstrated the pro‐oxidant action of glucose primarily via generation of mitochondrial ROS from endothelial cells (Nishikawa et al. 2000). Furthermore, it was demonstrated that these ROS induced PKC activation. Although the induction of mitochondrial ROS in response to glucose was subsequently confirmed in mesangial cells (Kiritoshi et al. 2003; Coughlan et al. 2009) and thus this pathway is considered to pertain not only to vascular but also to diabetic renal complications, the link to subsequent PKC activation has been less well characterized. Although ROS appear to activate PKC (Giorgi et al. 2010), oxidative stress may also play a role in the intracellular localization of PKC. For example, in mouse fibroblasts, oxidative stress triggers translocation of both PKC‐*α* and ‐*β* from the cytosol to the membrane (Giorgi et al. 2010). With increasing data linking intrarenal ROS to PKC activation and secondly Nox4 as a major source of renal ROS, particularly in the diabetic state, we measured PKC expression and activity in Nox4 KO mice. We demonstrated a diabetes‐induced increase in PKC activity in all renal cortical fractions examined. Importantly, this activation of PKC was ameliorated in the absence of Nox4. More specifically, protein expression of PKC‐*α* in membranes and in mitochondria was also attenuated in the diabetic Nox4 KO mice.

To further delineate the link between Nox4 and PKC, we assessed PKC‐*α* and ‐*β* expression in Nox4 KO mice in the presence and absence of diabetes as well as evaluating putative downstream effects of both PKC isoforms. Menne et al. (2006) have previously demonstrated that PKC‐*α* KO mice have reduced VEGF and increased nephrin expression in the context of diabetes. Indeed, in this study, we demonstrated that the diabetes‐induced increase in VEGF was attenuated in Nox4 KO mice associated with a normalization of nephrin expression and decreased expression of PKC‐*α*. Thus, it appears that Nox4 is a key upstream event in the pathogenesis of diabetic nephropathy with subsequent activation of PKC including PKC‐*α* leading to increased albuminuria as a result of increased VEGF and decreased nephrin expression.

In several other studies (Ohshiro et al. 2006; Meier et al. 2007), it was shown that diabetic PKC‐*β* knockout mice had reduced expression of TGF*β* in association with reduced renal fibrosis without effects on albuminuria. The diabetic Nox4 KO mice showed decreased PKC‐*β* expression in association with reduced TGF*β*1 activity and decreased matrix accumulation as well as reduced collagen IV and fibronectin accumulation. Thus, Nox4 deletion leads to reduced expression of both PKC‐*α* and ‐*β* isoforms. Further studies are warranted to interpret if the functional and structural effects of Nox4 inhibition could be at least in part as a result of inhibition of two key PKC isoforms. This hypothesis is further supported by recent important findings by Menne et al. (2013) who showed that diabetic PKC‐*α*/*β* double knockout (dKO) mice showed reduced albuminuria and VEGF expression as well as normalization of nephrin expression in the setting of reduced renal fibrosis. Our study demonstrates that absence of Nox4 results in similar renoprotection as that observed in the PKC dKO mice suggesting that a pharmacological approach to reduce Nox4 expression would be a suitable therapeutic strategy to consider as a way of inhibiting multiple PKC isoforms and thus acting as a novel effective renoprotective approach in diabetes.

Diabetic nephropathy is an increasing unmet medical need resulting in an increasing burden to the healthcare system. This study has focused on exploring the potential implications of deleting a specific isoform of the NADPH oxidase enzyme, Nox4 on PKC‐*α* activation. Indeed, it appears that Nox4 activation is a major driving force for excess ROS generation and activation of certain PKC isoforms within the kidney, thereby promoting renal injury in diabetes. Deletion of Nox4 appears to be effective in reducing the burden of renal disease in diabetes, as reflected by reduced glomerular injury and thus represents a potentially important therapeutic target for diabetic nephropathy.

## Acknowledgments

The authors would like to thank Maryann Arnstein for her technical support and Kylie Gilbert for her assistance with the animals.

## Conflict of Interest

The authors have no conflict of interest.
